# The effects of radiofrequency radiation on mice fetus weight, length and tissues

**DOI:** 10.1016/j.dib.2018.06.107

**Published:** 2018-06-30

**Authors:** Iraj Alimohammadi, Azadeh Ashtarinezhad, Baharak Mohamadzadeh Asl, Batol Masruri, Nargess Moghadasi

**Affiliations:** a Department of Occupational Health, School of public Health, Iran University of Medical Sciences, Tehran, Iran; b Department of Pharmacology and Toxicology, Faculty of Pharmacy, Shahid Beheshti University of Medical Sciences, Tehran, Iran

**Keywords:** Radiofrequency radiation, Mice fetus tissues, Vitamin C, Fetal and developmental abnormalities

## Abstract

The public concern of harmful effects of radiofrequency radiation exposure, especially with rapid increase in the use of wireless and telecommunication devices, is increasing. Some studies show fetal and developmental abnormalities as the result of radiofrequency radiation exposure. We aimed to investigate possible teratogenic effects of radiofrequency in 915 MHz on mice fetus and protective role of vitamin C. 21 pregnant mice were divided into 3 groups. Control group was in normal condition without any stressor agent. Exposure group was exposed to 915 MHz RFR (8 h/day for 10 days) and 0.045 µw/cm^2^ power density. The exposure plus vitamin C group received 200 mg/kg vitamin C by gavage and was exposed to 915 MHz RFR (8 h/day for 10 days) and 0.045 µw/cm^2^ power density. The fetus weight, C-R length were measured by digital balance and caliper. Tissues were assessed after staining with H & E. Our results showed significant increase in fetus weight and C-R length and also enlarged liver, tail deformation in mice fetus in exposure group. Although usage of vitamin C caused significant decrease in mentioned parameters. The outcome of this study confirms the effects of radiofrequency radiation on growth parameters such as body weight, length and some tissues in mice fetuses and protective effect of vitamin C. However more studies on non-ionization radiation in different frequencies and severity, during pregnancy are needed to clarify the exact mechanisms of these changes and better protection.

**Specifications Table**

**Table:** 

Subject area	Occupational health
More specific subject area	Biochemistry, Toxicology
Type of data	Table, figure
How data was acquired	All tissues analyzed according to Haematoxylin and Eosin (H&E) method which is the common and standard staining method used in medical diagnosis. Histological study of fetus abnormalities was done by optical microscopy. Body weight, length and diameter were measured by digital balance and caliper.
Data format	Raw, analyzed
Experimental factors	Mice fetus growth parameters and tissues were assessed after exposure to radiofrequency radiation.
Experimental features	The body weight and crown-rump length of mice fetus and tissue abnormalities were determined.
Data source location	Tehran, Iran
Data accessibility	Data are reported in this article

**Value of data**

● The result of this study is useful for workers and users that are exposed to radiofrequency radiation as a physical agent.
● Investigation of radiofrequency radiation effects in organogenesis period during pregnancy and protective role of vitamin C as a water-soluble antioxidant in body tissues and fluids are the innovation of this study.
● These data showed changes of growth parameters and abnormalities in tissues as the result of radiofrequency radiation exposure which could be useful for some organization such as Ministry of Health and Medical Education to recognize possible risks in vulnerable groups like pregnant women and their embryos and protect them more effective.

## 1 Data

**Fig. 1:**
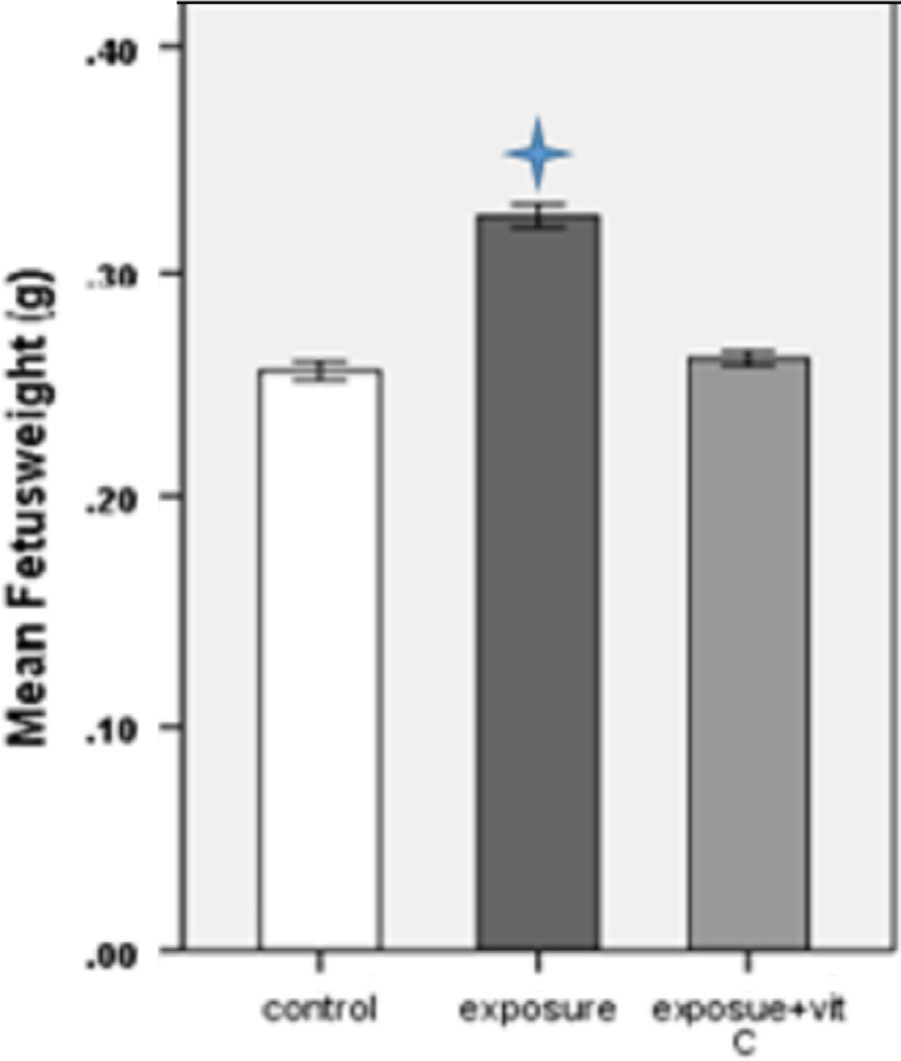
Comparison of mean weight of fetuses in 3 groups (5 fetus from each mice, 35 fetus per each group). *p<0.001 compared to control group.

**Fig. 2:**
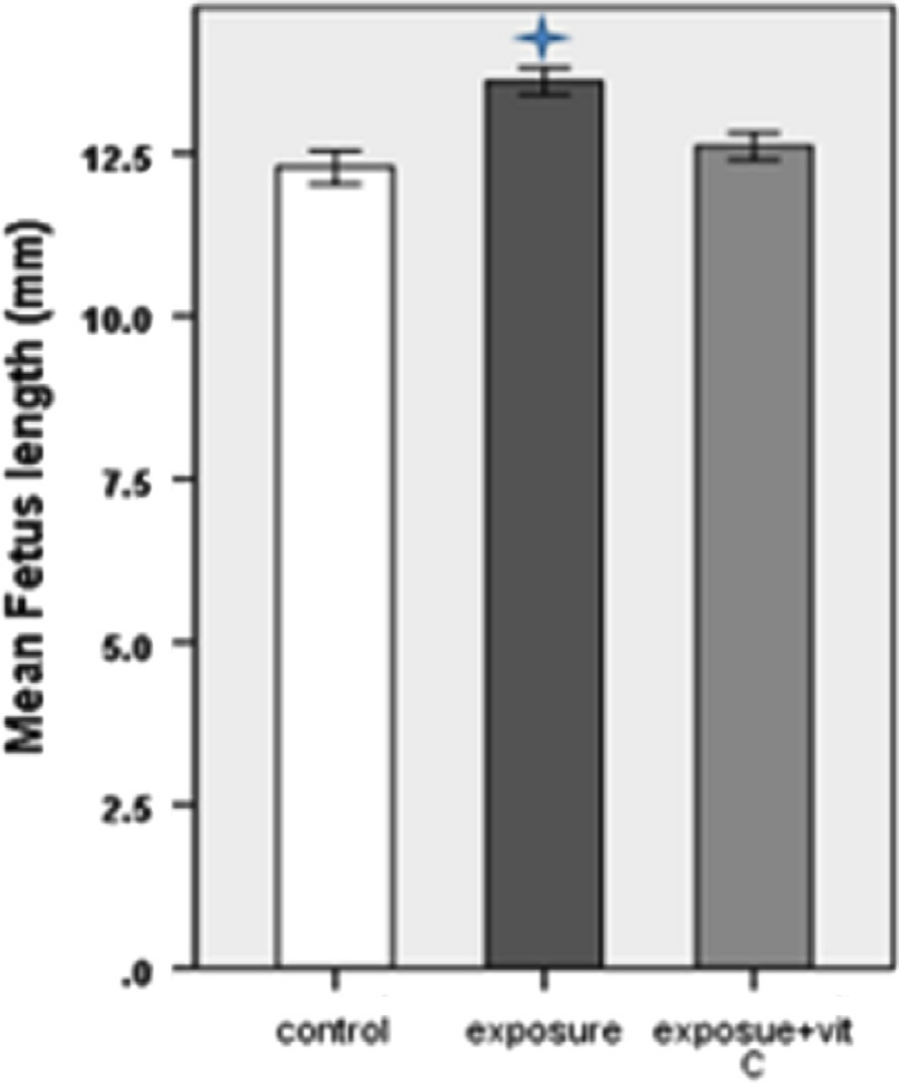
Comparison of mean length of fetus (C-R) in 3 groups (5 fetus from each mice, 35 fetus per each group). *p<0.001 compared to control group.

The fetus weight and length are shown in Table 1. Fetus weight and length are significantly increased in exposure group in compare with other groups. However, these parameters are increased in exposure plus vitamin C group in compare with control group but it was not statistically significant (Fig. 1, Fig. 2).

**Table 1:** The fetus weight and C-R length (Mean±SD) in 3 groups (35 fetus per each group).

Groups	Weight of fetus (mean± SD) (gr)	Length of fetus (mean±SD) (mm)
Control	0.25±0.009	12.28±0.61
Exposure	0.32±0.012	13.6±0.5
Exposure plus vitamin C	0.26±0.007	12.6±0.5

**Fig. 3:**
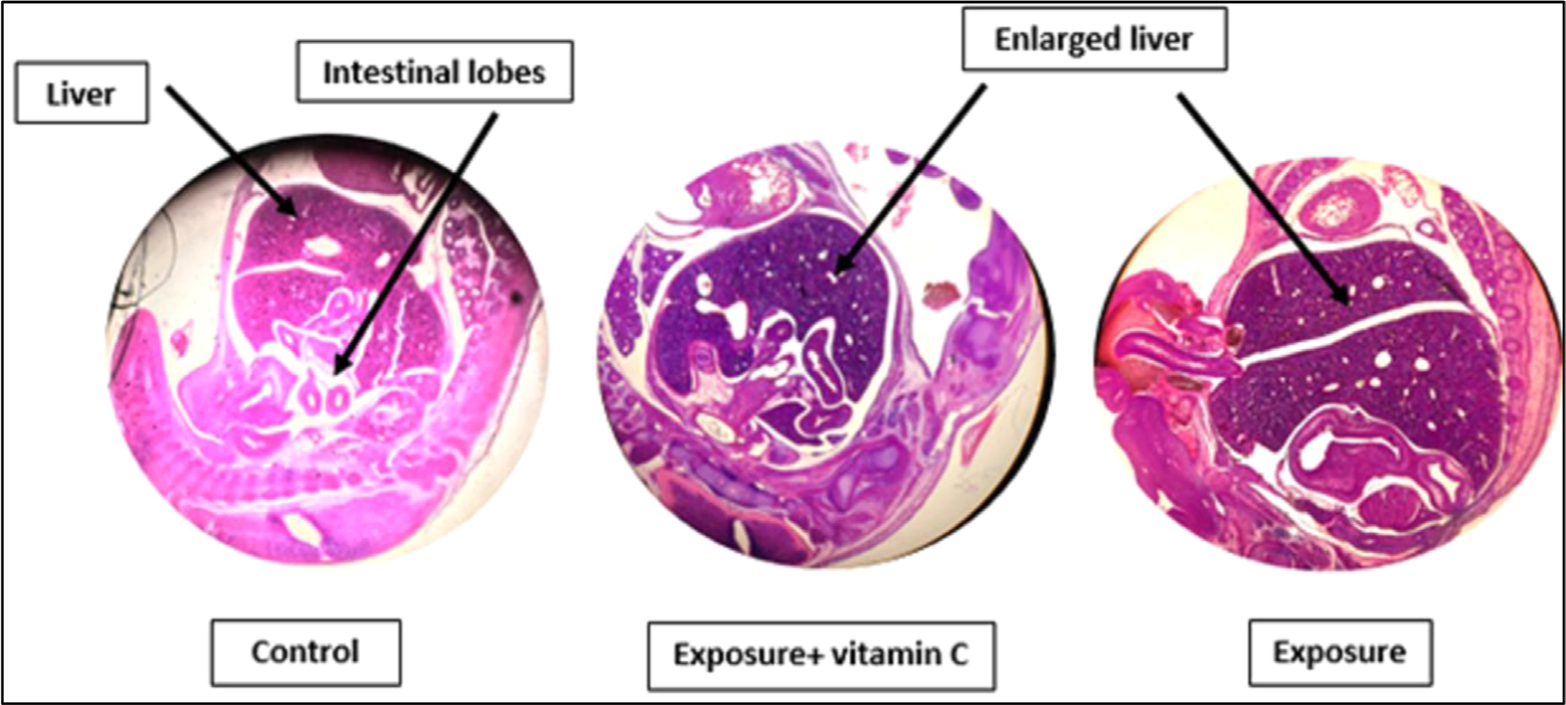
Comparison of the liver size and intestinal lobes location. The enlarged liver and abnormal intestinal lobes location in exposure with RFR in 915 MHz frequency and exposure with RFR in 915 MHz frequency plus vitamin C (200 mg/kg) groups is observed.

**Fig. 4:**
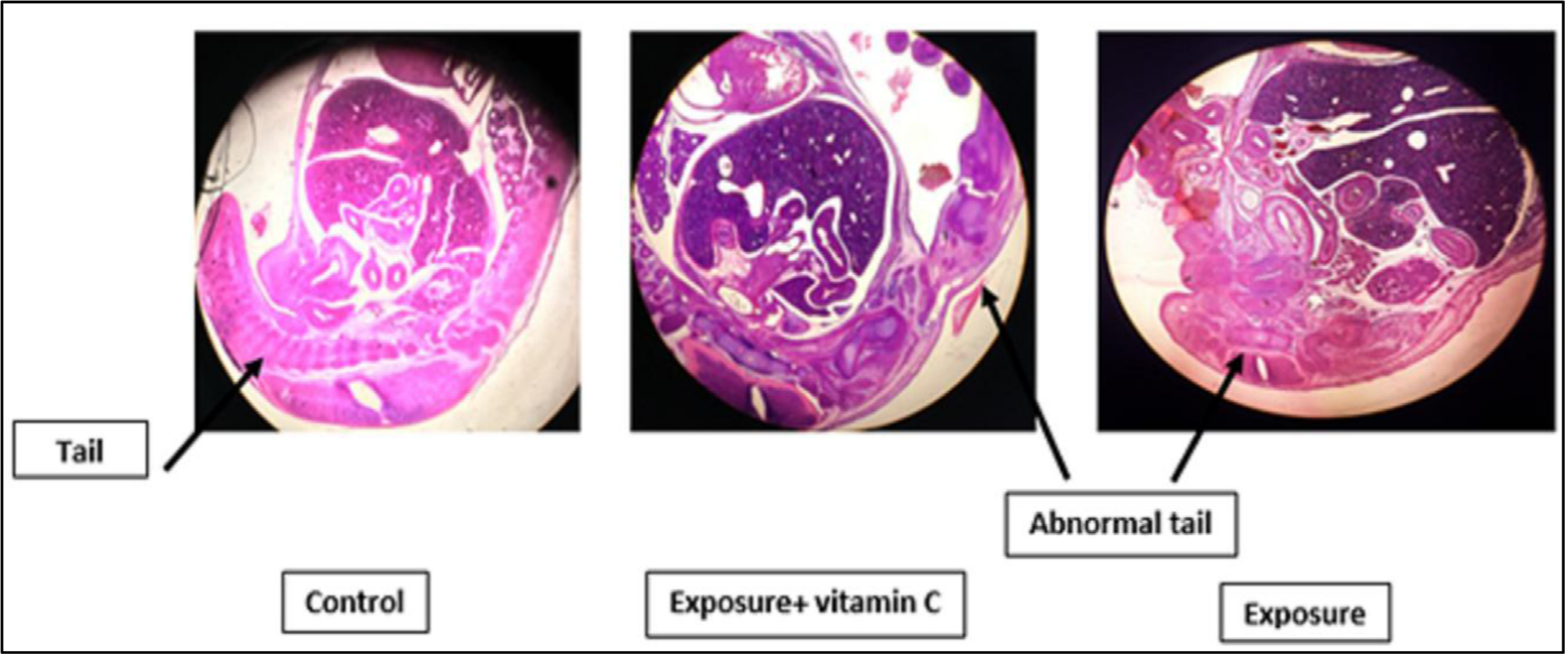
Comparison of tail form in experimental groups. The tail in exposure group with RFR in 915 MHz frequency and exposure group with RFR in 915 MHz frequency plus vitamin C (200 mg/kg) is not naturally formed as expected as control group.

As shown in Fig. 3, liver volume in exposure and exposure plus Vitamin C groups was larger than its normal expected size in the control group. The intestinal lobes were usually pulled to the abdomen in 15 days fetus but in this study, these lobes location in exposure and exposure plus Vitamin C groups was not normal respect to the control group. The tail in exposure group and exposure group plus vitamin C was not naturally formed as expected in control group (Fig. 4).

## 2 Experimental design, materials and method

### 2.1 Animal

21 adult NMRI mice (10–12 weeks) weighting 25–30 g were obtained from Experimental and Comparative Studies Center, Iran University of Medical Science, Tehran, Iran. The Animal Ethics Committee of Iran University of Medical Science has verification of the experimental protocol. The mice were kept in a standard situation with controlled light (12:12 h, light: dark), temperature (22 °C±2), changeable ventilation and relative humidity (40–50%) [1]. The animals were fed by standard diet (libitum and water). Mice were randomly mated overnight and pregnancy was detected by observing the vaginal plug. The day of detection of vaginal plug considered as zero day of pregnancy [2]. Pregnant mice were divided to 3 groups, 7 mice per each. Control group, exposure group and exposure plus vitamin C (200 mg/kg) group. The mice in control group were placed in polycarbonate cages with standard situation and received nothing [5]. The mice in exposure group were exposed to radiofrequency radiation (RFR) for 8 h/day from the first day of pregnancy to gestation day 10. The mice in third group were exposed to RFR for 8 h/day and received 200 mg/kg vitamin C by gavage from the first day to gestation day 10. Vitamin C powder (CAS Number: 50-81-7) was purchased from Sigma Aldrich.

### 2.2 Exposure set up

GSM-like signals in 915 MHz frequency were formed by using a radiofrequency signal generator with the integrated pulse modulation unit and horn antenna in a radiation chamber. The signals amplitude-modulated by rectangular pulses with a repetition frequency of 217 Hz. This RFR generator provided signals with 2 W and 0.045 µw/cm^2^ power density during exposure period. The signal was controlled by wave control device. The mice cage was put in 20 cm under the antenna. Mice moved freely and they received water and food as normal situation. During the experiment, the exposure setup and mice cages were in an isolated chamber to prevent interference of other radiations.

### 2.3 Sample preparation

Mice were sacrificed and dissected on day 15th of gestation and 5 fetus were selected from each mice. Measurements of weight of fetus and Crown-Rump (C-R) length were accomplished by digital balance and caliper, respectively. The fetuses fixed in Bouin׳s fixative solution for 18 h at room temperature, dehydrated (extraction of cells and tissues water) in graded ethanol series, and embedded in paraffin. Sections of fetuses were cut at thickness 10 µM by microtome [3]. Slices were placed on conventional glass slides for staining for Haematoxylin and Eosin (H&E) histological study of fetus abnormalities using optical microscopy. H&E is the common and standard staining method used in medical diagnosis [3], [4].

### 2.4 Microscopic investigation

Stained sections were assessed by optic microscope and development parameters and tissues were compared in groups [3], [6].

### 2.5 Statistical analysis

All the statistical analyzes were carried out using SPSS.22. The data were analyzed by one-way ANOVA test, Tukey and post hoc test. *P* value <0.05 was considered significant. The results were presented as mean±SD.
