# Supplementary material for: The effects of radiofrequency radiation on mice fetus weight, length and tissues
Source: Data Brief. 2018 Jun 30;19:2189–94. doi: 10.1016/j.dib.2018.06.107 (PMC6141437; doi:10.1016/j.dib.2018.06.107)
Supplement: Supplementary file 1 — Supplementary material [file mmc1.pdf]

Conflict of Interest

There isn't any Conflict of Interest.
